# Affordability, availability and acceptability barriers to health care for the chronically ill: Longitudinal case studies from South Africa

**DOI:** 10.1186/1472-6963-9-75

**Published:** 2009-05-09

**Authors:** Jane Goudge, Lucy Gilson, Steven Russell, Tebogo Gumede, Anne Mills

**Affiliations:** 1 Centre for Health Policy, School of Public Health, University of Witwatersrand, Johannesburg, South Africa; 2 Health Policy Unit, London School of Hygiene and Tropical Medicine, London, UK; 3 School of Development Studies, University of East Anglia, Norwich, UK

## Abstract

**Background:**

There is an increasing burden of chronic illness in low and middle income countries, driven by TB/HIV, as well as non-communicable diseases. Few health systems are organized to meet the needs of chronically ill patients, and patients' perspectives on the difficulties of accessing care need to be better understood, particularly in poor resourced settings, to achieve this end. This paper describes the experience of poor households attempting to access chronic care in a rural area of South Africa.

**Methods:**

A household survey (n = 1446 individuals) was combined with qualitative longitudinal research that followed 30 case study households over 10 months. Illness narratives and diaries provided descriptive textual data of household interactions with the health system.

**Results:**

In the survey 74% of reported health problems were 'chronic', 48% of which had no treatment action taken in the previous month. Amongst the case study households, of the 34 cases of chronic illness, only 21 (62%) cases had an allopathic diagnosis and only 12 (35%) were receiving regular treatment. Livelihoods exhausted from previous illness and death, low income, and limited social networks, prevented consultation with monthly expenditure for repeated consultations as high as 60% of income. Interrupted drug supplies, insufficient clinical services at the clinic level necessitating referral, and a lack of ambulances further hampered access to care. Poor provider-patient interaction led to inadequate understanding of illness, inappropriate treatment action, 'healer shopping', and at times a break down in cooperation, with the patient 'giving up' on the public health system. However, productive patient-provider interactions not only facilitated appropriate treatment action but enabled patients to justify their need for financial assistance to family and neighbours, and so access care. In addition, patients and their families with understanding of a disease became a community resource drawn on to assist others.

**Conclusion:**

In strengthening the public sector it is important not only to improve drug supply chains, ambulance services, referral systems and clinical capacity at public clinics, and to address the financial constraints faced by the socially disadvantaged, but also to think through how providers can engage with patients in a way that strengthens the therapeutic alliance.

## Background

There is an increasing burden of chronic illness in low and middle income countries, driven by TB and HIV, as well as cardio-vascular disease and diabetes [1-3]. However, few health systems are organized to meet the needs of chronically ill patients [4], particularly poor patients who have limited resources with which to seek regular care [5]. As a result, low and middle income countries often fail to mitigate rising chronic disease burdens [6]. This paper describes the difficulties poor households face in accessing chronic care in a rural area of South Africa, with the aim of informing health policy debates as to how the health system might be better organized to meet the needs of patients with chronic illness.

The key barriers to care are unaffordable costs to households, weak availability of inputs and services, and poor acceptability (the appropriateness of the social interaction that accompanies care), collectively referred to as the access framework [5]. In low and middle income countries, patients often either do not seek care, or do so only when they have access to funds, thus affecting continuity of care. Shortage of health service inputs (staff, drugs, and equipment) often mean that appropriate care is not available [7]. Complex treatment seeking patterns ('healer shopping'), where a patient consults a variety of providers, can also prevent the provision of regular chronic care [8-10]. Effective chronic care requires productive interactions between informed and prepared patients and organized and well-equipped health care teams in the context of an informed and supportive community (as outlined in Wagner's Chronic Care Model [4]). If health systems are to be organized to reduce access barriers the patients' perspective on the difficulties of accessing care and 'healer shopping' needs to be better understood.

The South African Costs and Coping study (SACOCO), one of the few studies to combine both quantitative cross-sectional and qualitative longitudinal data on the interactions between poor households and the health system, has documented treatment patterns and explanatory processes determining treatment action (or non-action) from the perspective of household members. South Africa, with its high levels of chronic non-communicable diseases [2] and TB/HIV epidemics [11,12], provides a relevant case study to examine the problems patients face in accessing chronic care. The public health facilities provide care for common chronic illness such as TB, hypertension, diabetes, and asthma, Although the rollout of treatment for HIV had just begun at the time of the study, there were examples of HIV infected individuals obtaining regular treatment within the public sector. Various measures have been designed to improve access to care over the last 13 years such as a clinic building programme, free primary health care, exemptions for hospital fees for the poor, cash transfers, and a patients' rights charter. Unconditional cash transfers were available at the time of study for women over 60 and men over 65 years (Pension, at US$74 [R740 at an exchange rate of R10 = U$1] per month), the disabled or those with specific long term diseases such as TB (Disability Grant, US$74 per month), and children under 11 years old (Child Support Grant, US$17 per month, means tested). Despite these measures South Africa still struggles to deal adequately with chronic care (witness the emergence of multi-drug resistant TB due to the high number of patients failing to complete treatment).

This paper uses survey data to examine the extent of chronic illness and whether patients are receiving regular treatment in a rural district in South Africa. It then draws on qualitative data to examine the factors constraining and facilitating access to chronic care. Finally, possible actions within the health system to improve access to care are identified. Complementary analysis is available elsewhere on the costs of ill health faced by poor households, illness-related impoverishment and the extent of social protection from cost burdens [13,14]

## Methods

The research was conducted in 2004–5 in the MRC/Wits-Agincourt Health and Socio-Demographic Surveillance site (21 communities with 70,000 people) in Mpumalanga province, South Africa. Despite being classified as a rural area, population densities are high, but infrastructure, such as sanitation and road networks, is poor. Electricity is affordable to a minority, and unemployment of working age adults is high at 60% (personal communication from Rural Public Health and Health Transitions Research Unit, Agincourt). Social grants (old age pensions as well as disability and child support grants) are important sources of income, as well as remittances from migrant labourers. Between 1998–2003 the growing HIV/TB epidemic and cardio-vascular disease had contributed to a fall in average life expectancy from 72 to 60 years for women, and from 66 to 52 years for men [15]. Within the field site there is a complex health system with 6 public clinics, 2 health centres, numerous traditional and faith healers and stores selling common over-the-counter drugs. Beyond the field site in the nearby urban centres (20 km away) there are pharmacies, allopathic private doctors, and public hospitals.

A household survey (n = 280 households, 1446 individuals), stratified by socio-economic status using the surveillance census data as a sampling frame, collected information on illness occurrence, health seeking behavior, household expenditure, and assets owned by the household from two purposively selected communities. The sample size was calculated to ensure that the survey population would include at least 30 households experiencing considerable ill health. The calculation was based on percentage of households that had reported at least 2 people sick in the last utilization survey as part of the broader HDSS work, and percentage of households experiencing a death in the last census. Two contrasting communities were selected. One community was well-established with a clinic and a good transport network, both characteristics likely to facilitate access to care; the second was without a clinic and had a poorer transport network, characteristics likely to hamper access. Due to the difficulty of determining a specific illness from respondents' descriptions of symptoms, any illness that had persisted longer than a month was defined as chronic.

30 case study households, with chronic illness so defined and stratified by socio-economic status, were selected from the survey. Choosing 15 households from each of the 2 villages allowed for 3 households in each socio-economic strata defined by quintiles. Selection was based on morbidity and hospitalization in the last year reported in the survey. Households were also selected to ensure a variation in characteristics such as receipt of social grants, and number of people in the household, Table 1 provides demographic information on each of the case study households. These 30 households were followed over 10 months to generate in-depth data and understanding of household experiences. For each of the illnesses included in the analysis, table 2 provides either an allopathic diagnosis where reported by the respondent, or a description of the symptoms. Chronic illnesses in the case study households that were resolved within 2 months were excluded from the analysis.

**Table 1:** Demographic information of case study households

**Livelihood status**	**Household Pseudonym**	**Age of household head**	**Number of household members**	**Description of household structure**	**Chronic illness cases**	**Grants**
Vulnerable	Silinda	49	8	Husband and wife (30 yrs), 3 sons/daughters (30-24 yrs), daughter in law (24 yrs), 3 grandchildren (7-yrs)		2 child support grants

Highly vulnerable	Nzima	37	2	Husband and wife (30 yrs)	Khulekani HV1	

Highly vulnerable	Khosa	59	14	Mother, 9 sons/daughters (30 – 7 yrs), 4 grandchildren (4-1 yrs)	Essie HV4	1 disability, 3 child support grants

Highly vulnerable	Thobela	48	11	Husband and wife (47 yrs), 8 sons/daughters (27-12 yrs), 1 grandchild (8 yrs)	Bohani HV8, Sipho HV14	1 pension, 2 child support grant

Highly vulnerable	Whatty	unknown	9	Husband and wife (40 yrs), 7 children (20-5 yrs)		2 child support grants

Highly vulnerable	Nkuna	68	8	Husband and wife (53 yrs), 5 sons/daughters (24-11 yrs), 1 grandchild (6 yrs)	Florah HV5, Elphas HV13	

Highly vulnerable	Manzini	49	4	Husband and wife (47 yrs), 1 daughter (18 yrs), 1 grandchild (2 yrs)	Phumuzile HV7, Ernest HV15	1 child support grant

Highly vulnerable	Mkhonto	42	6	Husband and wife(36 yrs), 4 sons/daughters (11-1 yrs)	Kulani HV3	2 pensions, 1 child support grant

Vulnerable	Mafuyeka	48	10	Mother, 6 sons/daughters (27-10 yrs), 3 grandchildren (7-5 yrs)		3 child support grants

Highly vulnerable	Tshabetha	69	13	Husband and wife (53 yrs), 7 sons/daughters (32-15 yrs), 4 grandchildren (12-4 yrs)	Railinah HV2, Philemon HV9, Jafeth HV11, Lindiwe HV12	1 pension

Highly vulnerable	Mhlanga	20	8	6 brothers/sisters (20-7 yrs), 2 sons/daughters (1 yrs)	Decan HV10	2 child support grants

Highly vulnerable	Sibuyi	38	4	Mother, 3 children (17-9 yrs)	Esther HV 16	1 child support grant

Highly vulnerable	Mnisi	46	5	Mother, 3 sons/daughters (30-16 yrs), 1 grandchild (11 yrs)	Polile HV6	1 disability grant

Secure	Makukule	83	11	Husband and wife (80 yrs), 4 sons/daughters (39-24 yrs), 5 grandchildren (19-1 yrs)	Nonhlanhla S29, S34	2 child support grants

Secure	Sithole	unknown	14	Husband and wife (40 yrs), 5 sons/daughters (15-1 yrs), sister/brother-in-law, nephews/nieces		1 disability grant

Secure	Zitha	70	3	Mother, 2 sons (32 yrs, 25 yrs)	Phinias S30, Precious S33	1 pension

Secure	Ndlazi	54	14	Husband and wife (50 yrs), 7 sons/daughter (33-18 yrs), 4 grandchild (11-4 yrs)	Dorcus S31	

Vulnerable	Zitha	58	8	Mother, 4 sons/daughters (33-16 yrs), 3 grandchildren (16-1 yrs)		1 child support grant

Secure	Ngwenya	77	3	Mother, 1 grandchild (24 yrs), 1 great-grandchild (4 yrs)	Ruth S32	2 child support grants

Highly vulnerable	Dlamini	52	4	Father, 3 sons/daughters (22-18 yrs)		

Secure	Madonsela	73	12	Mother, son (54 yrs) and wife, daughter (35 yrs), 9 grandchildren (23-5 yrs)	Khensani S28, Sbusiso S35	1 pension, 1 child support grant

Vulnerable	Mathebula	50	13	Husband and wife (38 yrs), mother in law (85 yrs), 10 sons/daughters (26-1 yrs)	Nomsa V17	2 child support grants

Highly vulnerable	Godi	55	2	Husband and wife (51 yrs)		

Vulnerable	Mlambo	39	7	Mother, 2 sons/daughter (39-33 yrs), 3 grandchildren (10-2 yrs)		2 child support grants

Vulnerable	Magemezulu	56	6	Husband and wife (56 yrs), 2 sons/daughters (22 yrs, 18 yrs), 1 grandchild (14 yrs),	Johannes V22	1 child support grant

Vulnerable	Gumede	43	21	Husband and wife (27 yrs), 4 brothers/sisters (38-27 yrs) 4 sons/daughers (13-1 yrs), others	Lungile V18	1 child support grant

Vulnerable	Sondlana	46	7	Husband and wife (45 yrs), 5 sons/daughers (25-3 yrs)	Glory V19, Clifford V24	2 disability grants, 1 pension

Vulnerable	Ndubane	60	12	Husband and wife (54 yrs), 4 sons/daughters (28-13 yrs), 6 grandchildren (13-1 yrs)	Losta V20, Nkukueko V23, Freddy V26, Glory V27	1 disability grant

Vulnerable	Siwela	66	4	Mother, 1 daughter (41 yrs), 2 grandchildren (19 yrs, 15 yrs)	Vusi V21, Nancy V25	

**Table 2:** Chronic illness cases with reported diagnosis or symptoms

**Chronic illness case number**	**Pseudonym**	**Reported allopathic diagnosis**	**Description of symptoms provided by respondent, when could not provide an allopathic diagnosis**
HV 1	Khulekani		Diarhorrea, sores, weight loss

HV 2	Railinah		Itching sores, vomiting

HV 3	Kulani		Leg and hand become stiff; fingers won't straighten; terrible headache

HV 4	Essie		headache, numbness in figures, painful legs

HV 5	Florah		headache; body pains and swollen hands; painful eyes, painful throat; numbness in leg;

HV 6	Polile		Collapses, faints.

HV 7	Phumuzile		Chest problem

HV 8	Bohani	TB	

HV 9	Philemon	TB	

HV 10	Decan	TB	

HV 11	Jafeth	TB	

HV 12	Lindiwe	Hypertension	

HV 13	Elphas	Hypertension	

HV 14	Sipho	TB	

HV 15	Ernest	AIDS	

HV 16	Esther	Hole in heart	

V 17	Nomsa		Rashes and sores

V 18	Lungile		Sores around stomach

V 19	Glory		Cough, shingles, dizzies, stabbing pains in stomach

V 20	Losta		Swollen legs, whole body painful, sharp pain under right breast

*V *21	Vusi	TB	

V 22	Johannes	Stoke	

V 23	Nkukueko	Asthma	

V 24	Clifford	TB	

V 25	Nancy	Hypertension	

V 26	Freddy	Asthma	

V 27	Gale	Asthma	

S 28	Khensani		Chest and knees problem, sometimes dark shade over her eyes

S 29	Nonhlanhla		Heart beating, sweating, difficulty breathing, fainted

S 30	Phinias	Mental illness	

S 31	Dorcus	Hypertension	

S 32	Ruth	Hypertension	

S 33	Precious	Hypertension	

S 34	Nonhlanhla	Epilepsy	

S 35	Sbusiso	Epilepsy	

Household histories, illness narratives, and monthly illness diaries including respondents' descriptions of visits to health providers were employed to obtain information from case study households. The diaries, forms on which details of ill health and treatment seeking in the previous month were recorded by respondents, provided initial information to prompt greater description in a subsequent detailed interview. Such interviews were conducted at least monthly, but were often more frequent when more detailed interviews such as the life histories or illness narratives were required. Documenting household interactions with the health system over time allowed identification and explanation of treatment patterns. The interviews focused not only on individual patients but also on the role of other household members and the broader social network, who might influence illness explanations, health seeking behaviour and coping strategies. Ethical approval was granted by the University of Witwatersrand Medical Ethics Committee and the ethics committee of the London School of Hygiene and Tropical Medicine, informed consent was obtained from all respondents, and pseudonyms have been used to ensure confidentiality.

### Analysis

Survey households were first grouped into expenditure quintiles to allow comparisons across groups defined by socio-economic status, using the Pearson chi-squared test to identify statistically significant patterns where appropriate. A livelihood analysis, a multi-dimensional approach considering people's assets (physical, human, financial, and social capital) and vulnerability to shocks, in addition to income and expenditure, was applied to the 30 case study households [16]. Households were categorised into three livelihood groups: a) those having a *secure livelihood *and meeting basic needs (such as food, health care, school fees) relatively easily; b) those with a *vulnerable livelihood *and meeting basic needs most of the time but without a secure, steady income; and c) those with a *highly vulnerable livelihood *and regularly not meeting basic needs, surviving on small intermittent earnings, gifts and grants. This household categorisation was undertaken independently by two researchers and adjusted following discussion and further review by two other researchers. Table 3 shows the match between expenditure quintiles and livelihood status.

**Table 3:** Livelihood status of case study households by expenditure quintile from household survey

	**Expenditure quintiles (Monthly household expenditure range in brackets)**				
**Livelihood status**	**Poorest Quintile** **(US$0–8)**	**2** **(US$9–15)**	**3** **(US$16–22)**	**4** **(US$23–38)**	**5** **(US$38+)**

Highly vulnerable	9	2	2		No case

Vulnerable		5	3	2	study

Secure		2		5	households

Source: Case study & household survey data

#### Secure livelihood

These households had at least one member with a secure job as well as other sources of income: for example, a male worker, a partner running a successful small enterprise, or working children, and a social grant. Average household size was about 8 members. Five households were located in the fourth overall expenditure quintile (Table 3). They had relatively strong asset portfolios, particularly in human capital (adult children with tertiary or vocational education in secure employment, able to help parents and other family members if they became ill). They had more physical assets than other groups: better built houses with more furniture and electrical items (e.g. TV, fridge) and more livestock, including some cattle. The few with debts had incurred these for business or furniture investments, and were making regular payments.

##### Vulnerable livelihood

These households had fewer and less secure sources of income than the first group, often temporary or contract-based employment. At least one person was employed (e.g. school cleaner or cook), running a small enterprise (selling beer, clothes) or had a social grant. Average household size was about 10 people, usually with several unemployed adults and young children. Households in the group spanned income quintiles 2–4 (Table 3), but as half were in the second lowest quintile they sometimes struggled to meet food needs, and purchased food on credit from shops. Only two households had a member with a pension. In terms of human capital, adults were not well educated and the cost of post-school vocational training had excluded younger people from the few job opportunities available (such as a driver, waiter or game park tracker). Some had similar physical assets to those of the secure group, but others had not completed their houses or had fewer household and electrical goods, and only small livestock.

##### Highly vulnerable livelihood

No-one in these households was employed or earned a steady income. Five had no source of income and relied on gifts from relatives and neighbours; in some, a woman managed a very small enterprise (selling school snacks or firewood) that generated minimal and intermittent earnings; only three households had a member receiving a pension. Nine of the 13 households were in the poorest income quintile, with per capita incomes of US$8.00 per month or less (Table 3); two others in the second poorest quintile struggled to meet minimum daily food needs. Those without grants were not able to obtain food on credit from the local shop. This group had the most limited asset portfolios: fewer physical assets, and limited human capital. Adults either had little formal education, had lost employment due to previous illness, or could not work due to disability or current long-term illness, events that often had exhausted household livelihoods.

The intensive study of a small number of families over time enabled the research to explore experiences of seeking care for chronic illnesses, and how interaction with the health services shaped future engagement. The knowledge claims from case studies are often criticised on the grounds that the evidence is 'anecdotal' or 'unrepresentative'. But the case study approach was necessary to understand the processes that affected access to chronic care: the case studies could go beyond the identification of those not receiving regular treatment to reveal the processes operating between households and the health system that hamper access to care[17]. As case study data are not statistically representative but aim to strengthen understanding of social processes, sample size is of less concern than the depth of understanding generated[18,19].

## Results

### 1. Self-reported chronic ill health, non-consultation and regular treatment

#### Survey data

In the household survey 23% of individuals (339/1446) reported one or more health problems, and, of these health problems, 74% had lasted longer than one month ('chronic') (253 of 343). The poorest quintile showed a lower propensity to report a chronic health problem than the highest quintile, despite a greater level of self-reported 'poor' health status (Table 4).

**Table 4:** Frequency and percent of individuals with chronic illness, and poor or very poor health status, by quintile in the last month

	**Poorest**	**2**	**3**	**4**	**5**	**Total**	
Frequency & percent with poor or very poor health status	43 (13%)	24 (7%)	29 (8%)	29 (12%)	20 (11%)	145 (10%)	Pearson chi2 (16) = 47.3597, PR = 0.000

Frequency & percent with a chronic health problem	49 (15%)	31 (9%)	41 (12%)	28 (1%)	46 (26%)	195 (13%)	Pearson chi2 (4) = 27.3174, PR = 0.000

Total per quintile	337 (100%)	347 (100%)	335 (100%)	252 (100%)	175 (100%)	1446 (100%)	

Source: Household survey data

No treatment action was taken for 38% (129 of 343) of health problems in the last month. For one third of these problems the illness had either improved or was not considered serious enough to seek care, however, access barriers prevented consultation for two thirds of these problems [13]. Higher levels of non-consultation were associated with chronic (no action taken for 48% of illnesses) rather than acute (9%) illnesses.

Respondents were asked whether they had been told to take medication or special foods on a regular, on-going basis. The question encompassed not just allopathic medication, but any treatment action. Socio-economic status did not influence whether an action was taken, because free clinic care and no cost self-treatment action were available. 29% of chronic illness (74 of 253) had been prescribed a regular treatment. Among those prescribed a regular action, the higher income quintiles, and the very poorest quintile, were more likely to be prescribed regular allopathic medication. The three poorer quintiles were more likely to have been prescribed special foods (such as avoiding sour foods, drinking fridge water), or indigenous medicine (Table 5). Across all quintiles, however, only 73% (55 of 74) of those prescribed a regular treatment took that action.

**Table 5:** Type of regular prescribed treatment action for chronic illnesses by quintile

**Type of regular action**	**Poorest**	**2**	**3**	**4**	**5**	**Total**
No regular therapy prescribed	50 (75%)	24 (65%)	37 (65%)	26 (76%)	42 (72%)	179 (71%)

Allopathic medication	14 (21%)	8 (22%)	11 (19%)	8 (24%)	13 (23%)	54 (21%)

Indigenous medication	0	2 (6%)	5 (9%)	0	2 (3%)	9 (4%)

Special foods	1 (1%)	0	3 (5%)	0	0	4 (2%)

Check up	2 (3%)	0	1 (2%)	0	0	3 (1%)

Regular herbs	0	1 (3%)	0	0	0	1 (0%)

Blessed tea (from the Zionist Christian Church)	0	2 (5%)	0	0	1 (2%)	3 (1%)

Total	67 (100%)	37 (100%)	57 (100%)	34 (100%)	58 (100%)	258 (100%)

Source: Household survey data;

#### Case study data

**Figure 1:**
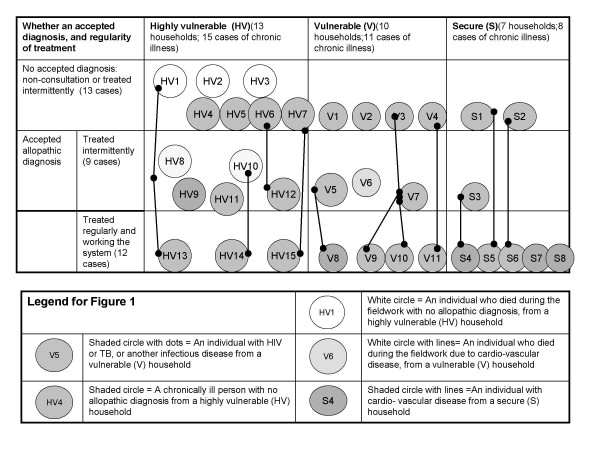
**Diagnosis and regular treatment of chronic illness by vulnerability of household**.

Figure 1 presents information on each of the 34 chronically ill case-study patients (each shown as a circle). 13 of the 34 cases (38%) had no diagnosis reported by respondents, whilst 21 (62%) cases had an allopathic diagnosis that the patient appeared to have accepted, as judged by the frequency the respondent used the diagnosis to describe the illness (without giving equal weight to alternative diagnoses). Only 12 of the 34 cases (35%) were receiving regular treatment.

The chronic cases were not evenly distributed across the 30 households. The lines between circles link chronic cases in the same household, showing that 23 of the chronic cases occurred in 10 of the households. Moreover, five of the six deaths occurred within the *highly vulnerable *households (marked as white circles with and without patterns in Figure 1). The *highly vulnerable *households also had more cases of HIV/TB and other infectious diseases (circles with dots), than the *vulnerable *or *secure *households. In contrast, the diagnosed cardio-vascular problems tended to be in the *secure *group (circles with lines), although, given respondents' descriptions of symptoms, it is likely that there were undiagnosed cases in the *highly vulnerable *group.

### 2. Barriers to accessing chronic care

#### 2.1. Inability to pay for the costs of seeking chronic treatment

##### Highly vulnerable households

Half (6/13) of the *highly vulnerable *households had no source of income and depended on gifts from family and neighbours, so regular health care consultation was very difficult. *"At the clinic we were told to take her to hospital. The problem was that we did not have money for transport" (Mother of Polile, Case HV5)*. As a result 13 of the 16 chronic cases (Fig 1) in the *highly vulnerable *group sought treatment at best intermittently and 4 of these 16 cases either hardly consulted at all or relied on self-treatment (*HV2 Khulekani, HV5 Polile, HV7 Phumuzile & HV9 Lindiwe*). Lindiwe and Khulekani's stories show how a combination of factors – unemployment or low grant income, livelihoods exhausted from illness and death, multiple illnesses, and limited social networks – prevented consultation:

Both Lindiwe's husband and daughter had been employed but both had died in the last year. With no social grant or employment income, Lindiwe (53 years) was now dependant on gifts from neighbours and friends to look after a family of 10, and as a result she had insufficient resources to seek care for her own chronic illness (*Case HV9*). Khulekani (37 years) had no source of income as he had lost his job due to ill health. With no relatives to assist, he couldn't afford to seek care, and subsequently died (*Case HV*2) (From field notes)

Obtaining an exemption from hospital fees was difficult in practice. Although all except two chronically ill patients were eligible for exemptions at public hospitals due to unemployment, fees were incurred for a third of public hospital visits (32 out of 90 visits) during the 10 months of fieldwork. Patients are required to provide proof of unemployment to obtain an exemption but obtaining the necessary paper incurs transport costs to the appropriate office.

Nonetheless, 3 of the 15 *highly vulnerable *chronic cases had obtained regular treatment and their symptoms appeared to be under control despite poverty (*Cases HV13, HV14, & HV15*). The key enabling factors were financial assistance from friends or relatives, government grants, and exemptions from public hospital fees. A pension or disability grant ensured access to an exemption as the receipt of a grant was sufficient proof of eligibility.

Esther (38 years) had no source of income and 3 children to support. However, she had a strong social network, with parents who ensured that she sought care. With their help she was able to secure a disability grant, and meet her own health care costs as well as other basic needs for the household. Due to the grant Esther was also able to obtain an exemption at the public hospital, and the cost of her monthly hospital visit amounted to 3–10% of household income (*Case HV13)*. (From field notes)

For those with no income, dependant on gifts from the family, the cost burden of monthly hospital care could be much higher. For example Ernest had to go repeatedly to hospital due to HIV, and the resulting cost burden fluctuated between 6–60% of monthly household expenditure, depending on how many times he was required to go, and whether he was too ill to travel by public transport (*Case HV15*). Without formal social support, other highly vulnerable households failed to obtain regular care.

##### Vulnerable and secure households

Although income was unpredictable or insufficient at times, *vulnerable households *were generally able to meet basic needs. All the 11 chronic cases from *vulnerable *households had sought care in the past, or were able to do so during fieldwork. The 8 cases in the *secure *group had sufficient resources to seek care during fieldwork. Phosiwe (*Case S4*) went regularly to hospital for her check up and to collect her hypertension tablets. The trip amounted to 4% of her monthly income.

#### 2.2 Limited availability of the inputs and services required for chronic care

Sufficient resources to seek care did not necessarily result in regular treatment and control of symptoms because of health system weaknesses and the unavailability of inputs and services required for chronic care. For example, only 4 of the 11 chronic cases in *vulnerable *households, and 5 out of 8 chronic illnesses in the *secure *households, were treated regularly (Fig 1). Weaknesses were of various types.

##### Clinical weaknesses in diagnosing and prescribing at clinics

The chronically ill respondents diagnosed with TB, high blood pressure, as well as HIV, had all attended a public hospital to commence treatment. For example, Ernest (*Case HV15*) visited the district hospital 4 times in the first month of his illness, firstly to treat his sores, secondly, for a TB test, and thirdly to obtain his TB results and have an HIV test. On his fourth visit his results were not ready and he was told to come back in 2 weeks. With each visit he had to pay transport costs and a consultation fee. For *highly vulnerable *and *vulnerable *households, repeat visits generated cost burdens amounting to 30–50% of monthly income (*Cases HV15, HV13, V4*), unaffordable without gifts from social networks[14]. For example, Decan (a 7 year old boy from a highly *vulnerable *household, *Case HV11*) had been unable to complete a course of TB treatment because of his mother's death. Although he was told he had to return to hospital to start a new course, his family did not take him because regular trips to hospital were unaffordable.

Patients with hypertension had to attend a hospital to obtain a confirmed diagnosis and appropriate prescription. After several months, or in some cases years of monthly visits to a public hospital, patients might be referred downwards to a primary care clinic so that they could collect their medication locally (Nancy *Case V8 *& Phosiwe *Case S4*). Elphas (*Case HV12*) was the one exception. After shopping around at different primary care clinics for several months, causing considerable delay, a nurse at his local clinic provided treatment without a hospital visit.

##### Interrupted drug supplies

Respondents complained that public clinics repeatedly ran out of drugs. For a *highly vulnerable *household such as Elphas's (*Case HV12*), the regular stock outs at his closest clinic led to 'shopping around', non-consultation and self-treatment, rather than wasting funds on transport for a fruitless trip to his local clinic. High blood pressure patients from *secure *households also faced regular drug shortages. As a result Ruth (*Case S8*) took a sample of her pills to the local chemist who sold her some without a prescription, and Phosiwe (*Case S4*) regularly returned to the district hospital to ensure she had the necessary supply of pills. In comparison, Elphas (*Case HV12) *from a *highly vulnerable *household who had more complex symptoms and an unclear diagnosis did not have the funds to go the chemist or to visit the hospital.

##### Weaknesses in the referral system

Referrals between public clinics and hospitals were common. The general pattern was initial identification of a chronic problem at the clinic, diagnosis and prescription at a hospital, and then either continued treatment at the hospital or referral to the clinic. Across the three livelihood groups, there were more successful referrals than failures. A variety of reasons explain the failures that did occur. Most common were the lack of an ambulance, or household inability to pay for transport and hospital fees. In one case differing diagnoses by the clinic and the hospital led to a failure of communication between the two leaving the patient confused as to where she should go for subsequent treatment (*Case V3 Losta*). In another, the necessary paperwork was not completed and when the family tried to trace a patient they were told that she had been discharged, when in fact she had been referred to a hospital further away *(Case V2 Nomsa)*. In two cases, the patients returned home without instructions to return to either hospital or clinic, despite continuing ill-health (*Cases V4 Glory & V5 Vusi*). Patients in this setting appeared relatively 'unempowered', unlikely to ask questions to clarify what to do next and likely to get 'lost' and give up. In particular, *highly vulnerable *households seemed less likely to take alternative action. For example Decan's siblings just continued to ask for TB treatment from the clinic (*Case HV10*), and Lindiwe resorted to not consulting and the use of herbs to control her symptoms (*Case HV9)*. In the *vulnerable *group, Nomsa turned to private doctors after a failed referral (*Case V2*), and Glory and Losta resorted to self-treatment and faith healers (*Cases V4 &V3*).

##### Inadequate ambulance services or lack of other subsidized transport

The access barriers for Decan (*Case HV11) *and others from *highly vulnerable *households included the transport costs of getting to hospital. Sipho (*Case HV14*) had been unable to complete a previous course of TB medication due to transport costs, and during the fieldwork became critically ill. An ambulance was not available to take him to hospital or to return him back to the clinic after his inpatient stay. On the first occasion all the drivers were attending a meeting; on the second, there was no ambulance in a suitable condition to transport patients. In contrast, patients from the *secure *group were able to pay the taxi fare to hospital, or use a relative's car. Thus, Phosiwe, Dorries, Nonhlanhla, and Sbusisio's mother (*Cases S4, S5, S6, S7*) all traveled to hospital on a regular basis to collect medication.

##### Tracing non-attending patients

For most chronic illnesses, regular attendance at a facility is crucial, and tracing non-attending patients is a necessary, but difficult task. The impact of failure to follow up with patients can be seen within the 30 case study households. Within the year prior to the start of fieldwork, there were 6 identified cases of TB that had not been cured, 4 of which had resulted in death. The difficulties in following up patients are significant, given staff shortages, lack of a system for reimbursing nurses for transport costs, and the difficulty of tracing patients. Despite these problems there were two cases of nurses going out of their way to reach out to patients experiencing difficulties in accessing care. In one case a nurse was worried about Sipho (TB 23 years old) and so visited his mother so that she could give appropriate advice (*Case HV14*). In the second case, the nurse visited Ruth (HBP 77 years old) to ask her to return to the clinic to collect her high blood pressure pills (*Case S8*).

#### 2.3 Unproductive patient-provider interactions and poor acceptability

For 13 of the 34 illnesses, respondents could not explain their illness, and did not have, or had not accepted, an allopathic diagnosis, despite seeking care, often more than once, at a public health facility (Fig 1). The case below shows how the lack of a clear diagnosis combined with problematic patient-provider interactions could lead to inappropriate treatment action or no action.

Kulani (an 11-year-old boy) had had difficulty breathing for several years. At one monthly visit his mother said he looked as if he had had a stroke as he was unable to straighten his fingers. The following month he fell from a sofa with a *'terrible headache'*, after which his leg and hand became stiff. His mother explained that neighbours said the illness was caused by *vukulu *(when social norms have been broken by borrowing items from the husband's relatives) while others said he had a stroke. After the fall the family consulted a prophet, a traditional healer and a clinic, which referred him initially to a local hospital, from where he was referred to a regional hospital. No family member was allowed to accompany him in the ambulance to either hospital, and with no funds for transport, the family could not talk to a doctor. They did not appear to have explanation for his illness or knowledge as to what was appropriate subsequent action. When Kulani returned in poorer health, his family saw the hospital treatment as a failure, consulted a faith healer rather than return to hospital, and the child died soon afterwards. *(Case HV3)*. (From field notes)

Without sufficient knowledge of their condition or treatment, some patients switched numerous times between healers ('healer shopping'), unclear as to who could provide relief.

In November, during an episode of shingles, Glory (45 years) visited a traditional healer, a Zionist Christian church prophet (ZCC), and 2 public clinics. In March with severe headache, dizziness, vomiting, and diarrhea, she became unconscious and unable to move for 3 days. Her husband took her to a ZCC prophet, who said it was caused by witchcraft, but he couldn't cure her. Her husband insisted she visit another faith healer. Over the next few months she continued taking blessed tea from the ZCC, and began to feel stronger. In June she began to cough and consulted a local clinic. Although clinic staff asked many questions, they gave her no explanation for her poor health. The cough continued after she had completed treatment from the clinic, and so she continued with the 'blessed' tea from the ZCC, but didn't return to the clinic. In July as the stomach cramps and chest pains became worse and were accompanied by numbness in her fingers, she visited a second (different) clinic. Although the staff treated her well, the clinic had run out of medication, and she was advised to go to the pharmacy. But Glory had no money left as her funds had been spent on a taxi fare to attend the clinic, and she returned home without medication (*Case V3*). (From field notes)

Glory's costly and unproductive shopping around between providers contrasted sharply with her husband's, Clifford. Clifford (*Case V11*) completed his course of TB treatment primarily because Glory reminded him to take his medication, collect his repeat prescription, and insisted he go back to hospital after he had prematurely stopped taking his pills. Glory clearly understood in this case the need to return to the same facility and complete treatment, but either a lack of a diagnosis, or the stigma associated with any diagnosis that had been given to her, prevented her from returning to the same facility.

Lunghile on the other hand, doubted the effectiveness of the treatment for his chronic illness, because it did not lead to cure.

Lunghile (43 years) had recurring sores around his waist, which '*seemed as if it was about to stop after a visit to the hospital*,' but only to return after the treatment had finished. "*So I don't know whether the medication doesn't have the power to kill this illness, or maybe it is not the right one*.' The respondent was not given enough information to be able to have clear expectations of what the treatment could achieve, and what subsequent actions were appropriate. During the 10 months he did not return to hospital to obtain further treatment to control his symptoms, despite his continuing ill health, explaining he wanted to cure his illness rather than just control the symptoms. (*Case V2*)

Despite its importance, effective communication by a provider is not a simple task. The following case illustrates this:

In December Ernest (49 years) was diagnosed as HIV+. He received a counseling session on living positively with HIV that he relayed in detail in the field interview. Ernest's openness about his status, and his disclosure to his family, suggested Ernest had accepted his HIV status. In July the field notes record: 'He told me that they changed his treatment. They even explained that he has another type of illness not HIV. He told me that they gave him tablets for the burning inside. "*I even thought that if they could have listened to me about how I was feeling, they shouldn't have given me those tablets that are for the viruses. If they could have given me the ones that they are giving me now I was going to be a much better person. I didn't say anything because I felt happy when they changed my treatment. When I took them I feel much better than when I was taking the other drugs*". (*Case HV15) *(From field notes)

Although Ernest had initially accepted his status, assisted by a thorough counseling session, several months later, due to confusing messages from health providers, he believed he had another illness.

When patient-provider interactions were productive, they not only enabled the patient to take the appropriate action, but also had two important additional effects. First, with sufficient understanding of the problem, and convinced of the efficacy of treatment, *highly vulnerable *households were able to explain and justify their need for financial support to members of their social network, enabling access to care. Second, patients and their families gained considerable experience of their disease and became a community resource that the health system could draw on to assist other patients. Below, Sipho's story *(Case HV14) *is contrasted with that of Jafeth (*Case HV11*) to illustrate these effects.

Sipho's household had no income, other than gifts from family and friends, due to the recent death of his father. Sipho (23 years old) had been treated for TB last year, but had defaulted on treatment. When he attended a clinic, the nurse thought he was hiding something, and so visited his home to talk to his mother. After an inpatient stay he had to attend the clinic for daily injections for 3 months. His mother had sufficient understanding to explain and justify their need for financial assistance. As a result friends and relatives provided approximately 300 rand a month, in a community where the average per capita income was 260 rand a month. Later on the clinic allowed the family to collect his pills when he had to travel to find work to support the family. Subsequently the mother became a volunteer in a local TB DOTS group, and gave informational and emotional support to others, as it had been given to her. (*Case HV14*)

Jafeth (11 years old) had had an uncompleted course of treatment for TB. He was taken to a second hospital where TB was not diagnosed but he was treated for other illnesses. Due to his continuing symptoms he was taken to a traditional healer. A neighbour advised the mother to take him back to the original hospital. The mother did so, but the nurses berated her for taking so long to return. The mother, humiliated, lied saying the child's parents were not at home, and that she was a neighbour who had come to the child's rescue. After returning home, the child was sent to his grandmother's to be looked after. (*Lindiwe's son, Case HV10*)

The humiliation experienced by Jafeth's mother did not 'empower' her to be of assistance to others. Instead she felt unable to ensure her son's return to health and this responsibility was passed to her mother. In contrast, the knowledge Sipho's mother gained from interaction with the nurses 'empowered' her to become a resource within the community.

### 3 The effects of combined access barriers

In most cases patients faced a combination of two or more of the access barriers distinguished above. In particular, patients who had more prolonged conditions with complex symptoms often had fewer resources, and were unable to take alternative action when faced with health system weaknesses. The combination of inability to pay the costs of seeking regular care, health system weaknesses, and unproductive interactions could lead to a breakdown in cooperation and trust between provider and patients. In the case described below, hospital staff did not understand the constraints Vusi faced due to poverty, and their unsympathetic treatment combined with weaknesses in the provision of care led Vusi to give up on public facilities.

Vusi (41 years) contracted TB while at school, but with insufficient income, had been unable to complete various courses of treatment. At 30 years old she was cured of TB, but the hospital continued to give her medication for continuing symptoms. When she explained these at the hospital – '*they (hospital nurses) said we have cured you of TB, we can't cure you twice*. *It seemed as if I was troubling them'*. On one visit to the hospital, the doctor recommended a blood test. She didn't inform the nurses, and they didn't do a test. On the subsequent visit, the nurses refused to let her join the queue to see the doctor without the test. Several times Vusi joined the queue, but then would be made to sit out on the side. Eventually the test was done. On returning for her results a few weeks later, she found the nurses hadn't sent off the bloods. They took the test again. On the next visit, there was an outstanding debt on her account from recent visits that she was unable to pay. The hospital refused to give her the test results until the amount was paid, but a doctor intervened. Vusi had also attempted to obtain a disability grant available to those on TB treatment, but the social worker said she couldn't request a grant because the doctor hadn't completed the correct paper work. She was told to return on another day. Towards the end of the fieldwork Vusi had given up on the public health system, and was a regular attendee at the ZCC church and their treatment of 'blessed' tea, where she was encouraged her to give up her pills. (*Case V5*) (Field notes).

Vusi's story is one of provider irritation with a patient who defaulted due to poverty, who now suffers from symptoms resulting from 20 years of TB and treatment, and who does not always follow instructions. It is also a story of patient frustration with a health system that did not seem to acknowledge her continuing symptoms, and at times obstructed her attempts to obtain care and a social grant. The breakdown in cooperation led to a failure to deal with a chronic illness.

## Discussion

This paper presents survey data and in-depth case studies of patient interactions with health services, intended to explore from patients' perspectives the factors preventing or causing the breakdown of regular chronic care in a resource poor setting. Households were selected using the socio-economic profile of the population in the surveillance site to ensure that their experiences were typical of chronic patients within the broader area.

The findings reported here show health care is not being sought for a substantial proportion of chronic illnesses, with many of those who have sought care not receiving regular care. Poor case identification and under-treatment have been shown to be important in other South African studies. Only 46% of those in need are receiving anti-retroviral treatment for AIDS [20]; evidence from rural south Africa suggests the health service identifies only 70% of TB cases [21]. Internationally, evidence shows high levels of mortality due to uncontrolled chronic disease. A recent study comparing data across 23 low and middle income countries reported death rates from chronic diseases 54% higher for men, and 85% higher for women, than in high income countries [3].

Availability of chronic care services is a first, and obviously central, factor influencing identification of illness and access to care. Respondents in the study faced a series of problems that included: insufficient clinical services at the clinic level necessitating referral, interrupted drug supplies, referrals that were hampered by a lack of ambulances, and weaknesses in administrative processes. Various South African studies on the quality of care provided at public facilities for hypertension and diabetes, for example, identify similar factors: nurses with insufficient knowledge to treat a particular chronic condition [22-25], a lack of functional equipment (such as baumanometers, broad BP cuffs, or equipment of measure blood glucose levels) leading, for example, to hypertensive patients being referred to hospital to initiate treatment [22], medicine shortage [22,26,27], and inadequate patient record keeping[28]. A recent review of health services research on chronic care in South Africa also identified increasing patient numbers, acute staff shortages, short consultation times, poor communication between staff, and lack of continuity of care by the same doctor as barriers to providing effective service [29]. Internationally, studies report lack of medication[30], lack of adequate clinical care [31-34] as well as high workloads and poor doctor motivation [30].

Tackling the identified problems in the South African setting is likely to require strengthening clinical primary level services to reduce the need for hospital visits, as well as improving transport provision and drug supplies. Of particular significance is improving the processes (e.g. maintaining patient information systems) and resources (e.g. additional staff, travel costs for health workers) with which to follow up patients, and to understand and assist with the difficulties that patients face in obtaining access. In addition, poor human resource management, and failure to recruit and retain sufficient health workers in rural areas, constrain service provision[35]. Strategies such as task-shifting to staff with lower levels of clinical skills [36,37], and use of community health workers or expert patients [38,39], are likely to be important in enabling the health system to reach out to those struggling to obtain access to care.

Inability to pay is a second factor preventing access to chronic care, as repeated consultations for a chronic condition can be a costly expense for poor households. Livelihoods exhausted from previous illness and death, continuing multiple illnesses, very little or no income, and limited social networks to provide financial assistance, prevented consultation for highly vulnerable households. The findings show the monthly cost burdens for repeated trips can be exceptionally high. Those households with income, strong social networks, receiving social grants, or exemptions from public hospital fees were able to seek care regularly, incurring much lower cost burdens. Although there is a growing international literature on the affordability of heath care [40-44], as well as literature on the household impact of illness and death as a result of catastrophic diseases such as HIV[45], there is little published evidence on the cost burdens of recurring chronic care. In a review of studies on the economic burden of HIV, TB and malaria in low and middle income countries, the direct costs incurred due to TB, requiring regular chronic care, were considerably higher (8–20% of annual income) than the costs incurred as a result of malaria (2–3% of monthly income)[46]. The review showed the largest cost from HIV were those associated with death, indicating regular treatment was not commonly available. Disease specific studies from South Africa have broadly noted that the lack of finances was an impediment to regular clinic visits [47], and following a prescribed diet[48]. The cost of traveling to hospital was also found to be prohibitive, and consequently many patients ran out of medicines between hospital visits [48]. However, there are few detailed South African studies of the costs of chronic care.

Given the costs incurred as a result of repeated consultations for chronic care, policies that protect poor households from the financial burdens are crucial in facilitating access to care. Ensuring existing exemptions reach intended beneficiaries is a first step. Another would be to exempt all patients suffering from specific chronic diseases from all user fees [49]. Decentralising from hospitals to clinics, strengthening outreach activities, such as home visits by community health workers would also reduce the cost burdens faced by households significantly, as well as directly increasing access.

The third important influence over access is the acceptability of health services, defined as the social and cultural distance between health care systems and their users [5]. The findings of this study show over a third of the respondents with a chronic illness in the case study households did not have an allopathic diagnosis that they were able to report to fieldworkers, despite having sought care. This may have been due to a variety of reasons: a failure of the clinic staff to make a diagnosis, no explanation given to the patient, or the explanation was given but insufficient effort was made to ensure that the patient had absorbed and understood the information. Stigma may prevent the patient from absorbing, accepting or reporting the diagnosis. It may also prevent clear communication between health worker and patient. AIDS, still highly stigmatized, is a major cause of mortality in the field study area [15]. Other symptoms, such as sores, loss of the use of a limb, can be associated with indigenous illness and the breaking of cultural taboos, which are also stigmatized [50]. Whatever the reason for the lack of an accepted diagnosis, it is illustrative of the social and cultural gap between health workers and patients. A recent review of empirical literature from low and middle income countries[51] identified problems that shape patient and provider engagement: the patient's inability to exercise voice in medical care encounters; provider behaviours such as poor communication practices; and provider stereotyping of patients. Other constraining factors are the gap between indigenous and allopathic explanations of ill health, and perceived effectiveness of treatment and the possibility of cure. Although no study has looked at the acceptability of chronic care provided in South Africa, a few have examined patient satisfaction with care [52,53]. One analysis identified 'providers who let me talk', 'providers who listen to me', 'supportive providers', 'considerate providers', 'encouraging providers' as key determinants of the interpersonal dimension of patient satisfaction [52]. The manner in which nurses speak to patients, particularly the problem of verbal abuse, although not a frequent finding in this study, has been shown to be a substantial barrier to access, preventing patients from attending public clinics [54,55].

These findings demonstrate the need to achieve more productive interactions between patient and provider, as recommended by the Wagner model [4], through carefully considered efforts. Ensuring a patient can see the same health provider on return visits over a reasonable time period would enable continuity and potentially allow mutual understanding to develop. Health care workers also need to see it as part of their responsibility to provide a time and space for patients to exercise voice, to ask questions, and express the difficulties they face in accessing care, as outlined in the 'client-centred approach' [56]. However, in the South African context where morale and motivation are low among nurses, and working conditions poor [57,58], having empathy for others is difficult. The findings here show providers can, and do, play a crucial role in gradual empowerment of patients and their families. Sipho's case, in which the nurse made a home visit and established a rapport with the mother, illustrates this. However, re-orientating the organizational culture of the health system to encourage greater levels of caring behaviour is a difficult task [59]. Possible strategies are support groups that help health workers to deal with stress [60], employing members from social disadvantaged groups [56], and strengthening leadership and management, particularly human resource management [51,61].

## Conclusion

The detailed longitudinal data presented in this paper have shown the importance of all three access barriers (affordability, availability and acceptability) and the complex ways in which they compound each other. Availability issues are all the more acute if a household is grappling with affordability. Both the inability of households to pay for care and the lack of availability of services can generate unproductive patient-provider interactions, associated with unacceptable care. And unacceptable care can simply mean that households make no attempt to overcome other barriers. Indeed, unproductive interactions can dis-empower patients and their families, and can lead them to give up on the health system. Although many studies focus on one of the access components, relatively few have examined all of them and how they interact to prevent appropriate patient action in response to chronic illness [62].

However, the paper has also shown that productive interactions between provider and patient, leading to patient understanding of their illness and treatment, may enable appropriate patient action. Productive interactions, in addition, can generate additional positive alliances within the community, which in turn may provide financial resources to pay for treatment and related costs, as in Sipho's case. Productive interactions, although important to all those who are ill, are most important for the poorest because their limited resources and vulnerable livelihoods often prevent them from persisting in their search for illness understanding and relief from symptoms from the health system.

In strengthening the public sector it is important, therefore, not only to improve drug supply chains, ambulance services, referral systems, and clinical capacity at public clinics, but also to think through how providers can engage with patients in a way that strengthens the therapeutic alliance. Improvements in chronic care provision must be complemented by inter-sectoral action to address the financial constraints faced by socially disadvantaged groups. Without this complementary action the affordability barrier to access will remain, perpetuating conditions for poor acceptability of care.

## Competing interests

The authors declare that they have no competing interests.

## Authors' contributions

AM, SR, LG, JG – overall conceptualisation of project; SR, LG, TG and JG – design of data collection tools; TG, JG – data collection and analysis; JG – drafting paper; AM, SR, LG – commenting on written paper and assisting in re-drafting.

## Pre-publication history

The pre-publication history for this paper can be accessed here:


